# Effect of asiatic and ursolic acids on morphology, hydrophobicity, and adhesion of UPECs to uroepithelial cells

**DOI:** 10.1007/s12223-012-0205-7

**Published:** 2012-11-07

**Authors:** Wojnicz Dorota, Kicia Marta, Tichaczek-Goska Dorota

**Affiliations:** Department of Biology and Medical Parasitology, Wroclaw Medical University, Mikulicza-Radeckiego 9, 50-367 Wroclaw, Poland

## Abstract

Adhesion of bacteria to epithelial tissue is an essential step in the progression of the urinary tract infections. Reduction of virulence factors responsible for microbial attachment may help to decrease or inhibit colonization of the host organism by pathogens. In the age of increasing bacterial antibiotic resistance, more and more attention is being paid to the use of plants and/or their bioactive components in the prevention and treatment of human infections. Asiatic acid (AA) and ursolic acid (UA), two plant secondary metabolites, were used as potential antibacterial agents. The current study aimed to determine the possible impact of AA and UA on morphology, hydrophobicity, and adhesion of clinical uropathogenic *Escherichia coli* strains (UPEC) to the uroepithelial cells. Our work describes for the first time the effects exerted by AA and UA on virulence factors of UPECs. The impact of both acids on the cell surface hydrophobicity of the investigated strains was very weak. The results clearly show the influence of AA and UA on the presence of P fimbriae and curli fibers, morphology of the UPECs cells and their adhesion to epithelium; however, some differences between activities of AA and UA were found.

## Introduction

In most infectious diseases, the adherence of pathogenic organisms to the host tissues is an essential step of invasion leading to colonization (Pizarro-Cerda and Cossart 2006). Both specific (ligand-receptor like) and nonspecific (physicochemical) interactions may play an important role in the attachment ability of bacteria to the epithelial cells. Various outer membrane components such as fimbrial and afimbrial adhesions, flagella, proteins, and lipopolysaccharides are responsible for specific interactions between bacteria and the host cells. Bacterial adhesion is also governed by van der Waals forces, hydrogen bonding, electrostatic, and hydrophobic interactions. The role of adherence in the ability of uropathogenic *Escherichia coli* strains (UPECs) to induce urinary tract infections (UTIs) has been extensively studied (Mulvey 2002). Both P and type 1 fimbriae play a particular role in the adhesiveness of UPECs (Melican et al. 2011). Bacterial binding is also mediated by the hydrophobic interactions between uropathogenic rods and uroepithelial cell surfaces. It is known that adherence of bacteria to the epithelium is correlated with increasing cell surface hydrophobicity of the microorganism (Saralaya et al. 2004; Wojnicz and Jankowski 2007). Changes of the nature of bacterial cell surface could alter their adhesive capacity and thus reduce the spread of the infection in the human body.

Currently, many reports describe plants and their secondary metabolites as a promising source of potentially therapeutic agents. One of the most bioactive plant components are pentacyclic triterpenes (Chung et al. 2011). Their antimicrobial, anti-inflammatory, and antitumor activities have been reported (Cho et al. 2006; Fontanay et al. 2008; Ikeda et al. 2008; Filocamo et al. 2011). To our knowledge, triterpenes have not previously been studied for their bacterial anti-adhesive properties. Therefore, the purpose of our study was to determine the effect of AA and UA on the P fimbriae and curli fibers expression, cell surface hydrophobicity of uropathogenic *E*. *coli* strains and their ability to adhere to the human uroepithelium. Furthermore, the impact of both pentacyclic triterpenes on the cells morphology was assessed.

## Materials and methods

### Bacterial strains

Twenty uropathogenic *E*. *coli* strains were isolated from the urine specimens of patients with pyelonephritis, hospitalized in the Academic Clinical Centre of the Wrocław Medical University. *E*. *coli* identification was done by biochemical methods using the API-20E test kit (BioMérieux, Warsaw, Poland). The strains were maintained on Mueller–Hinton agar slopes (Oxoid) at 4 °C.

### Phylogenetic classification

Phylogenetic group was determined using primers specific for two genes (*chuA* and *yjaA*) and DNA fragment (*TspE4*.*C2*) according to the multiplex PCR method of Clermont at al. (2000), however the *yjaA* sequence was amplified separately. For this, the genomic DNA of each strain was isolated using GeneMATRIX Bacterial &Yeast Genomic DNA Purification Kit (EURx, Poland). The amplification products were separated by electrophoresis in a 2 % agarose gel. Gel images were visualized and analyzed using the Quantity One system (Bio-Rad). The strains were assigned to phylogenetic group B2 (*chuA*+, *yjaA*+) or D (*chuA*+, *yjaA*−) or B1 (*chuA*−, *TSPE4*.*C2*+) or A (*chuA*−, *TSPE4*.*C2*−).

### Antimicrobial agents

AA (purity, ≥97 %) and UA (purity, ≥90 %) were purchased from Sigma-Aldrich (Poznań, Poland). Stock solutions at a concentration of 10 mg/mL were prepared by dissolving acids in 96 % ethanol at 70 °C and stored at −20 °C. For all experiments, final concentrations of triterpenes were prepared by diluting the stock with Mueller–Hinton broth (MHB).

### Antimicrobial testing

The minimal inhibitory concentrations (MICs) of AA and UA were determined by the broth microdilution method recommended by the Clinical Laboratory Standard Institute (CLSI 2008). Briefly, the stock solutions (10 mg/mL) of triterpenes were dissolved in MHB to give the concentrations of 4,096 μg/mL and then diluted twofold to achieve the concentrations from 4 to 1,024 μg/mL. Then, 200 μL of each concentration was added in well (96-well microplate) and inoculated with the tested strains, yielding a bacterial density of 5 × 10^6^ CFU/mL. After 24 h incubation at 37 °C, the MIC was defined as the lowest concentration that inhibited bacterial growth. Each assay was repeated three times.

### Effect of AA and UA on P fimbriae expression

UPEC strains were incubated with AA and UA at concentrations of 10, 20, 30, 40, and 50 μg/mL for 24 h at 37 °C and next were washed three times in phosphate-buffered saline (PBS). Equal volumes of bacterial suspensions (0.5 McFarland) and 3 % solution of human erythrocytes with or without d-mannose were mixed to determine the ability of the tested strains to haemagglutination (Evans et al. 1980).

### Effect of AA and UA on curli fibers expression

*E*. *coli* strains were incubated with AA and UA (10–50 μg/mL) for 24 h at 37 °C. After incubation, bacteria were washed three times and next 10 μl of bacterial suspensions were inoculated onto YESCA agar plates containing congo red. Curli-producing *E*. *coli* bound to Congo red dye and formed red colonies, whereas curli-negative bacteria formed white colonies (Hammar et al. 1995).

### Effect of AA and UA on hydrophobicity of bacterial cells

UPEC strains were incubated with AA and UA at concentrations of 10, 20, 30, 40, and 50 μg/mL for 24 h at 37 °C. Following the incubation, bacterial cells were washed three times in PBS. After last centrifugation, the bacterial suspensions were diluted to obtain final optical density (measured at 470 nm) of 1.0. Untreated cells were assessed as a control. The salt aggregation test (SAT) of ammonium sulfate was used (Lindahl et al. 1981). The control and treated suspensions (20 μL) were mixed with a series of dilutions of ammonium sulfate (20 μL) ranging from 0 to 3.2 mol/L. The lowest concentration of ammonium sulfate at which bacteria aggregated was determined. Based on the SAT values, the strains were classified as: 0.1–0.2 mol/L, very strong hydrophobic; 0.4–1.0 mol/L, strong hydrophobic; 1.2–1.6 mol/L, hydrophobic; ≥1.8 mol/L, hydrophilic.

### Effect of AA and UA on adhesion to epithelial cells

The cell adhesion assay was performed essentially as described previously (Wojnicz et al. 2012). Human uroepithelial cells from fresh urine of nonbacteriuric females were resuspended in PBS to give 10^5^ cells per milliliter (Bürker chamber count). Bacteria were grown in MHB in the presence of 10–50 μg/mL AA and UA, harvested by centrifugation (4,000 rpm for 20 min), resuspended in PBS and adjusted to a concentration of 1.5 × 10^8^ CFU/mL. Equal volumes of epithelial cells and pentacyclic triterpene-treated bacterial suspensions were incubated for 1 h at 37 °C with shaking. Unattached bacteria were removed from the suspension by centrifugation (200 rpm for 20 min) and washing three times in PBS. The final pellets were air dried on glass slides and May–Grünwald stained. The attached bacteria on 40 separate cells were counted by direct light microscopy (Nikon Eclipse 400) and adherence was determined as the mean number of bacteria attached per cell. Control values were determined using epithelial cells mixed with bacteria without AA and UA (Jahanshahi et al. 2010).

### Effect of AA and UA on bacterial cell morphology

The strains were incubated at 37 °C for 24 h with AA and UA at concentrations of 50, 150, and 250 μg/mL. The bacterial samples were then washed three times in PBS. The final pellets were air dried on glass slides and Gram-stained and observed in Nikon Eclipse 400 microscope. The shape of bacterial cells and the length of the filaments and their proportions in the total number of microorganisms per 100 randomly observed bacteria were recorded. The microorganisms with length of 5–15 μm were classified as short filaments, those with >15 μm as long filaments. Experiments were done separately three times.

### Statistical analysis

All values are given as mean ± SD. The differences in adhesion and morphology between rods exposed to AA and UA and unexposed were analyzed by a *t* test for independent samples. All tests were analyzed at the significance level *P* < 0.05 using Statistica 7.1.

## Results

### Molecular characterization of bacterial strain

PCR assays revealed that the 20 *E*. *coli* isolates fell into two phylogenetic groups B2 (*n* = 16, 80 %) and D (*n* = 4, 20 %).

### Antibacterial activity

The MIC values of AA and UA against the 20 isolates of *E*. *coli* were high and distributed in a range from 512 μg/mL to>1,024 μg/mL.

### Effect of AA and UA on P fimbriae expression

All tested *E*. *coli* strains expressed P fimbriae. All concentrations of both acids caused the loss of the ability to agglutinate human erythrocytes. Results are shown in Table 1.

**Table 1:** The effect of AA and UA on the presence of P fimbriae and curli fibers in *E*. *coli* strains (*n* = 20)

	Concentration (μg/mL)	Presence of virulence factor (% of strains)	
		P fimbriae	Curli fibers
AA	0	100	100
	10	80	100
	20	65	100
	30	65	100
	40	60	80
	50	60	75
UA	0	100	100
	10	80	100
	20	65	100
	30	65	100
	40	65	95
	50	65	75

### Effect of AA and UA on curli fibers expression

All examined *E*. *coli* rods were curli-producing strains. As shown in Table 1 only the highest concentrations of AA and UA (40 and 50 μg/mL) affected the synthesis of curli fibers.

### Effect of AA and UA on hydrophobicity of bacterial cells

Of the 20 studied *E*. *coli* strains, four possessed very strong hydrophobic surface—they aggregated at 0.1–0.2 mol/L of ammonium sulfate. The cell surfaces of 11 strains were strongly hydrophobic exhibiting aggregation at 0.4–1.0 mol/L of ammonium sulfate. The rest of the strains displayed a hydrophilic nature. In the next stage of our study, we determined the effect of AA and UA at concentrations of 10, 20, 30, 40, and 50 μg/mL on the cell surface hydrophobicity of 15 hydrophobic strains. The change of the cells’ surface character from very strongly hydrophobic to strongly hydrophobic was observed exclusively at the highest of tested triterpene concentrations (50 μg/mL) in three cases after the treatment with UA and in two cases after exposure to AA.

### Effect of AA and UA on adhesion to epithelial cells

**Fig. 1:**
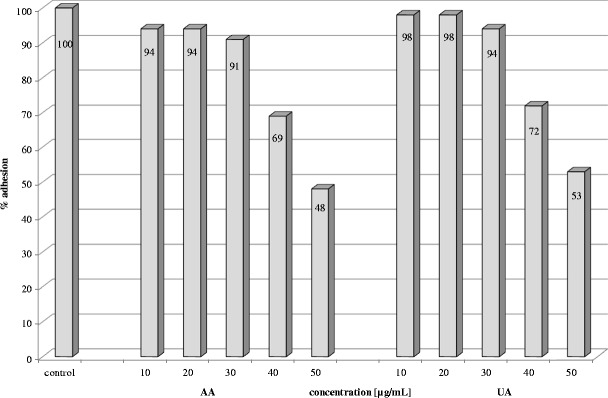
Effect of AA and UA on the adhesion of *E*. *coli* strain to the uroepithelial cells

**Fig. 2:**
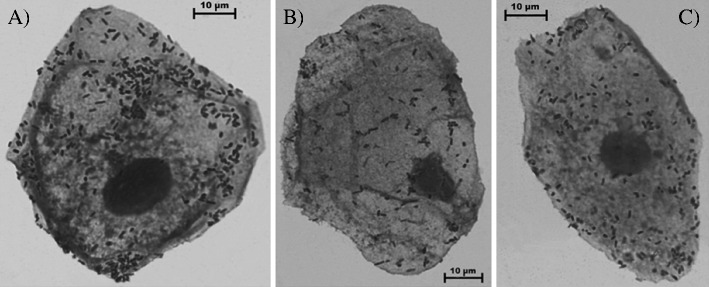
Adhesion of *E*. *coli* strain to the uroepithelial cell unexposed (**a**), exposed to 50 μg/mL AA (**b**), and 50 μg/mL UA (**c**). Magnification, ×1,000

The adhesion of 15 UPEC strains with very strongly hydrophobic and strongly hydrophobic cells surface, possessing P fimbriae and curli fibers, to the uroepithelial cells was determined. The results are shown in Figs. 1 and 2. The mean number of untreated bacterial cells attached to the one uroepithelial cell was 313.0 ± 27.7 (Fig. 2a). The adhesion of all strains was reduced following treatment with both AA and UA at concentrations of 40 and 50 μg/mL. The mean number of bacteria attached to the one epithelial cell was significantly reduced to 69 % (216.0 ± 19.2) and to 48 % (152.0 ± 16.7) after the treatment with 40 and 50 μg/mL AA, respectively (*P* < 0.05; Fig. 2b). The effect exerted by UA on the adhesion of UPECs to uroepitheliuim was slightly weaker. The mean number of bacteria attached to the one uroepithelial cell was reduced to 72 % (225.0 ± 11.8) and 53 % (166.0 ± 21.4) after incubation in 40 and 50 μg/mL AA, respectively (*P* < 0.05; Fig. 2c). These results were also statistically significant (*P* < 0.05). The changes in adhesion of bacteria treated with lower concentrations of acids were statistically insignificant.

### Effect of AA and UA on bacterial cell morphology

**Fig. 3:**
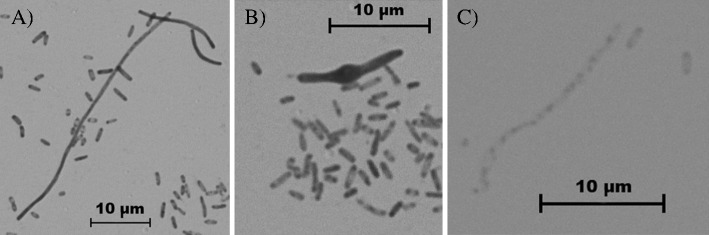
Morphological changes observed in *E*. *coli* strains grown in the presence of UA: **a** long and short filaments, **b** filament with mid-cell swellings, **c** ghost cell. Magnification, ×1,000

The control samples of the 20 investigated *E*. *coli* strains contained rods of normal length (96.1 %) and short filaments (3.9 %; Table 2). Only the exposure of bacteria to AA and UA at concentration of 250 μg/mL induced morphological changes. The lower concentrations of both triterpenes did not alter bacterial morphology. Significant changes in the shape of bacterial cells were observed after exposure to UA (*P* < 0.05). Microscopic analysis revealed the presence of long (40.45 %) and short filaments (10.9 %), ghost cells (2.35 %), and short filaments with mid-cell swellings (1.25 %; Fig. 3). In the UA-containing suspensions, the normal length bacterial cells (45.05 %) were also observed. AA had a much weaker impact on bacterial morphology. *E*. *coli* rods exposed to AA formed only short (6.15 %) and long filaments (2.95 %); neither “swollen” forms nor ghost cells were observed. The normal length bacteria accounted for as much as 90.9 % of the total cell number.

**Table 2:** Morphological changes observed in *E*. *coli* strains after incubation with AA and UA

	The mean number of cells per 100 randomly observed bacteria				
	Normal length (2–5 μm)	Short filaments (5–15 μm)	Long filaments (˃ 15 μm)	“Swollen” filaments	Ghost
Control	96.10 (±1.86)	3.90 (±1.86)	0	0	0
AA	90.90 (±2.15)	6.15 (±1.63)	2.95 (±1.28)	0	0
UA	45.05 (±6.00)	10.90 (±1.97)	40.45 (±4.70)	1.25 (±0.64)	2.35 (±1.18)

## Discussion

Adhesion of UPECs to the uroepithelium is a crucial step in the pathogenesis and colonization of the urinary tract. The hydrophobic interactions between bacteria and host tissues are important adhesion-promoting factors. Bacterial surface hydrophobicity is correlated with increased pathogenic potential (Dykes et al. 2003). It is well-documented that *E*. *coli* strains causing UTIs possess hydrophobic cell surfaces (Najar et al. 2007). We established that 15 out of 20 tested *E*. *coli* strains also possessed a hydrophobic character. It may confirm a significant role of this virulence factor among bacterial strains responsible for pyelonephritis. Results of research conducted by Sunman et al. (2001) and Raksha et al. (2003) also showed hydrophobic nature of the cell surface of *E*. *coli* isolated from patients with UTIs. It is known that the change of the bacterial cell surface from hydrophobic to hydrophilic correlates with the limited colonization of epithelial cells (Wojnicz and Jankowski 2007). The effects of various phyto-extracts on the bacterial cell surface hydrophobicity have been reported in several papers. These plant extracts exhibit modulating activity on the cell surface hydrophobicity of the microorganisms and thus potentially affect their pathogenicity (Barnabas and Nagarajan 1988; Nishino et al. 1987; Rauha et al. 2000; Dykes et al. 2003). For example, the aqueous extract of bearberry has been shown to alter the hydrophobicity of *E*. *coli* (Turi et al. 1999) and *Helicobacter pylori* (Anuuk et al. 1999). In another study, Nostro et al. (2004) found that surface hydrophobicity and adherence of *Streptococcus mutans* was reduced when bacteria were grown in the presence of the *Helichrysum italicum* extract.

Due to the absence of reports devoted to the effects of single phytocompounds on the bacterial cell surface hydrophobicity and adherence, we decided to investigate the impact of pentacyclic triterpenes (AA and UA) on these virulence factors. It is known that the biological activities of triterpenes may be related to the effect of these compounds on membranes, especially of the eukaryotic cells (Prades et al. 2011). This phenomenon is related to the structural similarity of pentacyclic triterpenes to cholesterol. These compounds could possibly be incorporated into the biomembranes instead of cholesterol and change their hydrophobic properties. Our study showed that neither AA nor UA have a significant effect on cells surface hydrophobicity. Such limited impact of AA and UA on the tested UPECs may probably be caused by the absence of the cholesterol in bacterial cells. Moreover, pentacyclic triterpenes can form micellar phases that could affect their incorporation into cell membrane and the ability to change the cell surface hydrophobicity (Rafat et al. 2008).

It is worth noting that, despite the weak antihydrophobic activity, both triterpenes significantly reduced the attachment of bacteria to urinary epithelial cells. Adhesion significantly decreased after treatment of bacteria with 40 and 50 μg/mL AA and UA. The mechanism of this inhibitory effect may probably be associated with suppression of the synthesis of the bacterial surface structures such as P fimbriae and curli fibers related to adhesion of rods to the host tissues. We cannot comprehensively discuss our results with respect to other reports because the data from other laboratories mainly describe the changes in the adhesiveness of bacteria treated with plant extracts or fruit juice. Well recognized is the adhesion-preventing activity of *Vaccinium macrocarpon* against *E*. *coli* and *H*. *pylori* (Johnson-White et al. 2006). Cunningham et al. (2004) and Foo et al. (2000) reported that cranberry proanthocyanidins are responsible for anti-adhesion of *H*. *pylori* and associated with urinary tract infections *E*. *coli* rods. Yamanaka et al. (2004) noticed that cranberry juice decreased adsorption of oral streptococci to saliva-coated hydroxyapatite beads. Similarly, extracts of *H*. *italicum*, *Mikania laevigata*, *Mikania glomerata*, *Syzygium aromaticum*, *Piper betle*, and *Piper guajava* showed positive anti-adherence activity to the saliva-coated glass surface against oral streptococci (Nostro et al. 2004; Yatsuda et al. 2005; Rahim and Khan 2006; Razak and Rahim 2003). Only one paper describes the impact of UA on bacterial adherence properties (Moodley et al. 2011). Decreased adhesion to polystyrene surfaces was noticed for *E*. *coli* and *S*. *aureus* incubated in subinhibitory (subMIC), MIC and suprainhibitory (supraMIC) concentrations of UA. For *Pseudomonas aeruginosa*, decreased adhesion was observed only after exposure to subMIC and MIC of UA, while increased adhesion was observed at supraMIC concentration of this triterpene. In contrast, the adhesion of *Staphylococcus saprophyticus* to polystyrene surfaces was increased after treatment of bacteria in subMIC but decreased after treatment in MIC and supraMIC concentrations of UA. Moodley et al. (2011) noticed that UA demonstrated the greatest ability to prevent bacterial colonization in comparison to oleanolic acid and methyl oleanolate. It was also interesting that the adhesion of *Klebsiella pneumoniae* was increased after exposure to all concentrations of UA (Moodley et al. 2011).

In addition to changes in bacterial cell surface hydrophobicity, the alteration of cell morphology can decrease the adhesion of pathogens to host cells. Untreated *E*. *coli* appear rod-shaped with the lengths ranging between 2 and 5 μm. We observed that the exposure of these organisms to the AA and UA resulted in morphological abnormalities. Formation of filaments, ghost cells, and mid-cell swellings forms were recorded. The results were dependent on type of triterpene used. All altered bacterial forms listed above were observed only after the treatment of UPECs with subMIC of UA. Incubation of bacteria with AA led to formation of long filaments, not observed in control samples; however, the percentage of them was very low. Kurek et al. (2010) also investigated the impact of pentacyclic triterpenes UA and oleanolic acid (OA) on the morphology and peptidoglycan synthesis of *Listeria monocytogenes*. They noticed that the length of bacterial cells was reduced. Szakiel et al. (2008) observed that *Bacillus megaterium* incubated with OA also became visibly shorter. In contrast, *E*. *coli* cells appeared several fold longer after the exposure to this acid. Bacterial filamentation is often observed as a result of DNA damage, inhibition of replication or alteration of FtsZ protein that is key to bacterial cell division (Justice et al. 2006). Morphological alterations observed in *E*. *coli* cells after their exposure to AA and UA may indicate that triterpenes can penetrate into the bacterial cells and interact with DNA, proteins involved in the septum formation or affect the replication process.

**Fig. 4:**
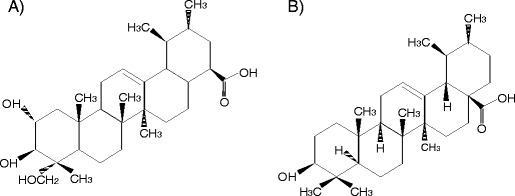
Chemical structures of AA (**a**) and UA (**b**)

Based on the differential effects exerted on *E*. *coli* by UA and AA, it is possible that they may arise from the differences in the chemical structure of these compounds (Fig. 4). Research conducted by Wen et al. (2005) on the relationship between structure and activity of pentacyclic triterpenes showed the A-ring structure to have a significant impact on biological activity. Despite the structural similarities of the triterpenes in the other rings, the A-ring in AA and UA is very different, with two additional hydroxyl groups in AA which could possibly affect bacterial length and shape.

In conclusion, interest in natural products has increased quite significantly in the past decade. Medicinal plants as well as their secondary metabolites have been assessed for possible bioactive agents for prevention of different human infections. Despite this, there are still many unknowns in this field, requiring further in-depth research.
